# Specimen Copies

**Published:** 1877-01-20

**Authors:** 


SPECIMEN COPIES.—We send this number to many medical brethren whose names are not on our list. We respectfully ask them to subscribe, and to indicate it by remitting us $2 at once. If you decline, please return the copy to

R. C. WORD, M. D.,
Business Manager.

